# Green leafy vegetables in diets with a 25:1 omega-6/omega-3 fatty acid ratio modify the erythrocyte fatty acid profile of spontaneously hypertensive rats

**DOI:** 10.1186/s12944-018-0723-7

**Published:** 2018-06-15

**Authors:** Melissa Johnson, Ralphenia D. Pace, Wendell H. McElhenney

**Affiliations:** 1 Department of Food and Nutritional Sciences, Tuskegee University, Tuskegee, AL 36088 USA; 2 Department of Agricultural and Environmental Sciences, Tuskegee University, Tuskegee, AL 36088 USA

**Keywords:** Erythrocyte, Collard greens, Fatty acid profile, Omega-6/omega-3 fatty acid ratio, Purslane, Spontaneously hypertensive rat, Sweet potato greens

## Abstract

**Background:**

In addition to the actual composition of the diet (i.e. nutrient composition, food groups), the omega-6/omega-3 fatty acid ratio has been demonstrated to influence the tissue fatty acid profile and subsequently the risk for cardiovascular and other diseases. Likewise, the consumption of green leafy vegetables (GLVs) may favorably reduce the risks associated with disease. Although an ~ 3:1 omega-6/omega-3 fatty acid ratio (ω-6/ω-3 FAR) is recommended, the typical American diet has an ~ 25:1 ω-6/ω-3 FAR. Previous research affirms the ability of collard greens (CG), purslane (PL), and sweet potato greens (SPG) to improve the hepatic profile of spontaneously hypertensive rats (SHRs). The aim of the present study was to determine the influence of GLVs, incorporated (4%) into diets with a 25:1 ω-6/ω-3 FAR, on the erythrocyte fatty acid profile of male SHRs.

**Methods:**

SHRs (*N* = 50) were randomly assigned to one of five dietary groups – standardized control (AIN-76A), Control (25:1 ω-6/ω-3 FAR), CG (25:1 ω-6/ω-3 FAR + 4% CG), PL (25:1 ω-6/ω-3 FAR + 4% PL) or SPG (25:1 ω-6/ω-3 FAR + 4% SPG). Following 6 weeks consumption of diets, SHRs erythrocyte fatty acid profiles were determined by gas-liquid chromatography.

**Results:**

Significantly lower percentages of total saturated fatty acids (*p* < 0.05) and greater percentages of polyunsaturated fatty acids were present among SHR erythrocytes following the consumption of diets containing CG, PL and SPG. Total polyunsaturated fatty acids were greatest among SHRs consuming diets containing purslane.

**Conclusions:**

The present study demonstrates the ability of GLVs to mitigate the potential effects of an elevated ω-6/ω-3 FAR, which may contribute to an atherogenic fatty acid profile, inflammation and disease pathogenesis. Dietary recommendations for disease prevention should consider the inclusion of these GLVs, particularly among those consuming diets with an ω-6/ω-3 FAR that may promote disease.

## Background

Epidemiological and clinical evidence affirms that the consumption of diets with elevated omega-6/omega-3 fatty acid ratios (ω-6/ω-3 FARs) to be associated with an increased risk for hypertension, cardiovascular disease (CVD), diabetes and other chronic diseases [1–3]. Further, the dietary ω-6/ω-3 FAR has been demonstrated to influence tissue fatty acid compositions [4, 5]. Although an ~ 3:1 ω-6/ω-3 FAR is recommended, the typical American (i.e. Western) diet has an ~ 25:1 ω-6/ω-3 FAR [6, 7]. The excessive consumption of vegetable oils, processed foods and refined products, such as those observed in Western cultures, are believed to contribute to elevations in the dietary ω-6/ω-3 FAR [8, 9]. Conversely, plant-based diets, particularly those containing vegetables abundant in α-linolenic acid, have lower ω-6/ω-3 FARs [10] and are plentiful in antioxidant and bioactive compounds that have been associated with decrease risk for chronic disease [11–13].

Green, leafy vegetables (GLVs), rich of sources of antioxidants and bioactive compounds, have been demonstrated to improve antioxidant status and reduce the risks associated with disease [14]. Further, dietary patterns that promote the increased consumption of GLVs, such as the Mediterranean diet, may be beneficial in reducing the risks associated with disease pathogenesis [15–18]. In addition, the Dietary Approaches to Stop Hypertension (DASH) diet endorses the consumption of plants commonly found in the African American diet such as collard greens and sweet potatoes, for the reduction of the risks associated with hypertension and other chronic diseases [19–22].

Collard greens (*Brassica oleracea*), a traditional GLV with the diet of Americans living in the southern United States, in addition to purslane (*Portulaca oleracea*) and sweet potato greens (*Ipomoea batatas* L.), novel GLVs within the diet, are potent dietary reservoirs of antioxidant and bioactive compounds that may decrease disease risk [23, 24]. Previous research has demonstrated the ability of collard greens, purslane and sweet potato greens to favorable modify the hepatic fatty acid profile of spontaneously hypertensive rats after 4 weeks consumption [25]. The aim of the present research study was to evaluate the influence of collard greens (CG), purslane (PL) and sweet potato greens (SPG), supplemented into diets with a 25:1 ω-6/ω-3 FAR, on the erythrocyte fatty acid profiles of male spontaneously hypertensive rats.

## Methods

### Animals and diets

Fifty (*N* = 50) male spontaneously hypertensive rats (SHRs), 4 weeks of age, were housed individually in clear polypropylene cages (43x27x15cm), with temperature and relative humidity controlled at 70-72 °C and 50–55%, respectively. SHRs were maintained on a 12:12 h light-dark photoperiod cycle. Following a 10 day acclimation period, SHRs were randomly assigned to one of four experimental dietary groups with a 25:1 ω-6/ω-3 FAR: 1) Control, 2) 4% CG, 3) 4% PL, 4) 4% SPG; 10 SHRs were assigned to the standardized control dietary group and received the AIN-76A diet for the duration of the research study. SHRs consumed the diets for 6 weeks. The compositions of the experimental diets are presented in Table 1. Animals were paid fed based on the average previous day’s intake of SHRs consuming the experimental diets containing CG, PL and SPG. SHRs were allowed to consume water ad libitum.

**Table 1:** Ingredient composition of standardized control and experimental diets fed to SHRs for 6 weeks^a^

	Dietary Group				
Ingredient (%)	AIN-76A	C	CG	PL	SPG
Sucrose	50.00	41.96	39.27	39.49	39.39
Casein (Vitamin Free)	20.00	18.00	16.82	16.53	16.68
Corn Starch	15.00	15.00	15.00	15.00	15.00
Powdered Cellulose	5.00	5.00	5.00	5.00	5.00
AIN-76 Mineral Mix	3.50	3.50	3.50	3.50	3.50
AIN-76 Vitamin Mix	1.00	1.00	1.00	1.00	1.00
DL-Methionine	0.30	0.30	0.30	0.30	0.30
Choline Bitartrate	0.20	0.20	0.20	0.20	0.20
Ethoxyquin^b^	0.00	0.00	0.00	0.00	0.00
Corn Oil	5.00	12.06	11.96	12.01	11.97
Soybean oil	–	2.91	2.88	2.89	2.89
Fish Oil	–	–	–	–	–
Cholesterol	–	0.07	0.07	0.07	0.07
Collard Greens	–	–	4.00	–	–
Purslane	–	–	–	4.00	–
Sweet potato Greens	–	–	–	–	4.00

^a^Diets formulated and manufactured by the Division of Land O’Lakes Purina Feed, LLC, Richmond, IN. C, control; *CG* collard greens, *PL* purslane; *SPG* sweet potato greens; ^b^Ethoxyquin content = 0.0010%

AIN-76A = AIN -76, standard rodent chow; C (control diet) = AIN-76A diet with a 25:1 ω-6/ω-3 FAR; CG = AIN-76A diet with a 25:1 ω-6/ω-3 FAR + 4% collard green powder; PL = AIN-76A diet with a 25:1 ω-6/ω-3 FAR + 4% purslane powder; SPG = AIN-76A diet with a 25:1 ω-6/ω-3 FAR + 4% sweet potato green powder

Following a 24 h fast animals were anesthetized using a Ketamine-Acepromazine combination cocktail and then euthanatized via over-inhalation of carbon dioxide. Blood was collected via cardiac puncture, collected in heparin-coated tubes and centrifuged at 2500 rpm at 10 °C for 30 min to separate plasma and erythrocytes. Following centrifugation, samples were stored at − 80 °C prior to analyses. Eight (*n* = 8) SHRs were randomly selected from each dietary group for the erythrocyte fatty acid profile analysis. The procedures involved in the care and use of the animals were approved by the Tuskegee University Animal Care and Use Committee.

### Erythrocyte fatty acid extraction

Erythrocyte fatty acid methyl esters (FAMEs) were prepared following transesterification with boron trifluoride (BF_3_, cat# 3–3021, 12% methanol, Supelco, Inc., Bellefonte, PA) using the procedures previously described by Masood et al. [26]. To approximately 0.01 g of SHR erythrocytes, 100 μl of nonadecanoic acid (C19:0, Nu-Chek Prep, Inc., Elysian, MN), dissolved in hexane (1.0 ml), and BF_3_ (1.0 ml) was added. Fatty acid methyl esters (FAMEs) were prepared by heating the mixture in a hot water bath at 55 °C for 90 min and subsequently placed in an ice bath for 5 min. Hexane (2.0 ml) and deionized water (1.0 ml) were added, Pyrex glass culture tubes were flushed with nitrogen and vortexed for 15 s. Following centrifugation at 2000 rpm for 5 min, the top organic layer, containing the FAMEs were collected and placed in gas chromatography (GC) vials for GC analysis. Samples were analyzed in duplicate.

### GC analysis of FAMEs

Erythrocyte FAMEs were isolated and quantified using a HP 6890 N network gas chromatograph system (Agilent Technologies, Santa Clara, CA) equipped with a HP 7683 series automated injector, flame ionization detector and a DB23 fused silica capillary high resolution gas chromatograph column (60 m, 0.25 mm, i.d., 0.25 μm film thickness, J&W Scientific, Folsom, CA). Data are expressed as percentages of total fatty acid.

### Statistical analysis

Statistical analyses were conducted using analysis of variance software (SAS Software, Cary, NC). Duncan’s post hoc procedures were performed to test if differences existed among SHRs consuming the different diets. Statistical significance was determined at *p* < 0.05.

## Results

Erythrocyte saturated fatty acid (SFA) concentrations (% total fatty acids) of SHRs consuming diets with a 25:1 ω-6/ω-3 FAR are presented in Table 2. Erythrocyte SFA concentrations were less among SHRs consuming diets containing CG (41.72 ± 2.71), PL (39.65 ± 1.41) and SPG (38.63 ± 0.80) in comparison to the standardized control (71.82 ± 3.43) and control (45.25 ± 2.36) diets. Palmitic acid was the most abundant erythrocyte SFA among SHRs, with SHRs consuming diets containing CG (24.71± 1.60), PL (23.77± 0.90) and SPG (23.05 ± 0.46) - demonstrating lower percentages of this fatty acid in comparison to the standardized control (60.05 ± 5.47; *p* < 0.05) and control (27.08± 1.61) diets.

**Table 2:** SHR erythrocyte saturated fatty acid composition (%total fatty acids) following the consumption of diets with a 25:1 ω-6/ω-3 FAR for 6 weeks^§^

		Dietary Group				
Fatty acid	Structure	AIN-76A	C	CG	PL	SPG
Capric	C10:0	nd	nd	nd	nd	nd
Undecanoic	C11:0	nd	nd	nd	nd	nd
Lauric	C12:0	0.24 ± 0.00^a^	0.43 ± 0.22^ab^	0.06 ± 0.01^a^	0.12 ± 0.04^ab^	0.16 ± 0.06^b^
Tridecyclic	C13:0	nd	nd	nd	nd	nd
Myristic	C14:0	0.17 ± 0.02^a^	0.23 ± 0.05^ab^	0.15 ± 0.03^a^	0.20 ± 0.03^ab^	0.29 ± 0.04^b^
Pentadecanoic	C15:0	0.12 ± 0.01^a^	0.14 ± 0.01^ab^	0.13 ± 0.01^ab^	0.17 ± 0.02^ab^	0.18 ± 0.01^b^
Palmitic	C16:0	60.08 ± 5.47^a^	27.08 ± 1.61^b^	24.71 ± 1.60^b^	23.77 ± 0.90^b^	23.05 ± 0.46^b^
Heptadecanoic	C17:0	nd	nd	nd	nd	nd
Stearic	C18:0	11.15 ± 2.80^a^	16.80 ± 1.04^b^	16.33 ± 1.05^b^	15.01 ± 0.52^ab^	14.52 ± 0.29^ab^
Arachidic	C20:0	nd	0.20 ± 0.01	nd	nd	nd
Behenic	C22:0	nd	nd	nd	nd	nd
Lignoceric	C24:0	nd	nd	nd	nd	nd
*Total SFAs*		*71.82 ± 3.43* ^a^	*45.25 ± 2.36* ^b^	*41.72 ± 2.71* ^b^	*39.65 ± 1.41* ^b^	*38.63 ± 0.80* ^b^

^§^Data are (expressed as) mean percentage ± SE. Values in the same row that do not share the same superscript letter are significantly different according to analysis of variance and Duncan’s post hoc procedures (*p* < .05); *nd* not detected

Total monounsaturated fatty acids (MUFAs) among SHRs consuming diets containing GLVs ranged from 13.11 ± 0.35 (CG) to 14.98 ± 0.70 (SPG) and were slightly less than consuming the control diet (15.10 ± 0.25) (Table 3). Oleic acid, the most abundant MUFA present, was greatest among SHRs assigned to the control (9.41 ± 0.33), CG (8.56 ± 0.35) and PL (8.55 ± 0.25) dietary groups. Significantly greater amounts of nervonic acid were present following the consumption of diets containing the GLVs in comparison to the standardized control diet; a slightly greater percentage of nervonic acid was present in the erythrocytes of SHRs consuming the control diet.

**Table 3:** SHR erythrocyte monounsaturated fatty acid composition (%total fatty acids) following the consumption of diets with a 25:1 ω-6/ω-3 FAR for 6 weeks^§^

		Dietary Group				
Fatty acid	Structure	AIN-76A	C	CG	PL	SPG
Undecenoic	C11:1	nd	nd	nd	nd	nd
Dodecenoic	C12:1	nd	nd	nd	nd	nd
Tridecanoic	C13:1	nd	nd	nd	nd	nd
Myristoleic	C14:1n5	nd	nd	nd	nd	nd
Pentadecenoic	C15:1n5	0.58 ± 0.08^a^	0.04 ± 0.00^b^	0.06 ± 0.00^b^	0.06 ± 0.01^b^	0.06 ± 0.00^b^
Palmitoleic	C16:1n7	0.28 ± 0.05^a^	0.14 ± 0.01^b^	0.16 ± 0.01^b^	0.15 ± 0.02^b^	0.10 ± 0.02^b^
Palmitelaidic	C16:1n7t	0.43 ± 0.04^a^	0.41 ± 0.05^a^	0.35 ± 0.05^a^	0.37 ± 0.04^a^	0.56 ± 0.03^b^
Heptadecenoic	C17:1n7	nd	nd	nd	nd	nd
Elaidic	C18:1n9t	nd	nd	nd	nd	nd
Vaccenic	C18:1n11c	nd	nd	nd	nd	nd
Trans-vaccenic	C18:1n7t	nd	nd	nd	nd	nd
Oleic	C18:1n9c	5.60 ± 0.61^a^	9.41 ± 0.33^c^	8.56 ± 0.35^bc^	8.55 ± 0.25^bc^	7.76 ± 0.23^b^
Cis-vaccenic	C18:1n7c	1.30 ± 0.17^a^	1.88 ± 0.08^b^	1.71 ± 0.07^b^	1.78 ± 0.06^b^	2.31 ± 0.09^c^
*cis*-5 Eicosenoic	C20:1n15	nd	0.31 ± 0.04	nd	nd	nd
*cis*-8-Eicosenoic	C20:1n12	nd	0.26 ± 0.03	nd	nd	nd
Eicosenoic	C20:1n9	0.07 ± 0.00^a^	0.26 ± 0.03^b^	0.23 ± 0.04^b^	0.19 ± 0.03^ab^	0.22 ± 0.02^b^
Erucic	C22:1n9	nd	nd	nd	nd	nd
Nervonic	C24:1n9	0.90 ± 0.20^a^	2.38 ± 0.23^b^	2.03 ± 0.19^b^	2.61 ± 0.41^b^	4.08 ± 0.40^c^
*Total MUFAs*		*9.09 ± 1.01* ^a^	*15.10 ± 0.25* ^c^	*13.11 ± 0.35* ^b^	*13.64 ± 0.39* ^bc^	*14.98 ± 0.70* ^c^

^§^Data are (expressed as) mean percentage ± SE. Values in the same row that do not share the same superscript letter are significantly different according to analysis of variance and Duncan’s post hoc procedures (*p* < .05); *nd* not detected

A significantly greater percentage of polyunsaturated fatty acids (PUFAs) were present in the erythrocytes of SHRs assigned to the control (40.30 ± 2.91), CG (45.50 ± 2.95), PL (46.70 ± 1.49) and SPG (46.51 ± 1.04) diets versus the standardized control diet (19.32 ± 2.81) (Table 4). In comparison to the control diet, slightly lower percentages of linoleic acid were present in the erythrocytes of SHRs consuming diets containing CG (8.69 ± 0.12) and PL (9.15 ± 0.19), while a significantly greater percentage of this fatty acid was present following the consumption of the diet containing SPG (10.3 ± 0.37). A greater percentage of α-linolenic acid was found in the erythrocytes of SHRs consuming diets containing CG (0.24 ± 0.07), PL (0.48 ± 0.22) and SPG (0.31± 0.02) in contrast to those consuming the standardized control and control diet.

**Table 4:** SHR erythrocyte polyunsaturated fatty acid composition (%total fatty acids) following the consumption of diets with a 25:1 ω-6/ω-3 FAR for 6 weeks^§^

		Dietary Group				
Fatty acid	Structure	AIN-76A	C	CG	PL	SPG
Linoelaidic	C18:2n6t	nd	nd	nd	nd	nd
Linoleic	C18:2n6c	3.68 ± 0.31^a^	9.26 ± 0.25^b^	8.69 ± 0.12^b^	9.15 ± 0.19^b^	10.31 ± 0.37^c^
γ-Linolenic	C18:3n6	0.23 ± 0.02^a^	0.63 ± 0.31^a^	8.48 ± 1.29^b^	6.43 ± 2.09^b^	5.07 ± 1.55^b^
α-Linolenic	C18:3n3	0.10 ± 0.04^a^	0.09 ± 0.02^a^	0.24 ± 0.07^a^	0.48 ± 0.22^a^	0.31 ± 0.02^a^
Eicosadienoic	C20:2n6	0.20 ± 0.03^a^	nd	0.51 ± 0.02^bc^	0.56 ± 0.03^c^	0.44 ± 0.03^b^
Eicosatrienoic	C20:3n6	0.19 ± 0.05^a^	0.43 ± 0.01^b^	0.40 ± 0.03^b^	0.40 ± 0.02^b^	0.57 ± 0.03^c^
Arachidonic	C20:4n6	12.25 ± 2.11^a^	22.65 ± 2.37^b^	22.41 ± 1.69^b^	22.09 ± 1.76^b^	21.67 ± 0.87^b^
Eicosatrienoic	C20:3n3	nd	0.16 ± 0.03^a^	0.17 ± 0.01^a^	nd	nd
Eicosapentaenoic	C20:5n3	nd	0.29 ± 0.07^a^	nd	nd	1.41 ± 0.23^b^
Docosadienoic	C22:2n6	nd	nd	nd	nd	nd
Docosatetraenoic	C22:4n6	1.30 ± 0.24^a^	2.26 ± 0.60^b^	2.02 ± 0.17 ^ab^	2.79 ± 0.34^b^	1.67 ± 0.33^ab^
Docosatrienoic	C22:3n3	0.78 ± 0.23^a^	1.12 ± 0.12^a^	0.84 ± 0.08^a^	1.07 *±* 0.18^a^	0.71 ± 0.12^a^
Docosapentaenoic	C22:5n3	nd	nd	nd	nd	nd
Docosahexaenoic	C22:6n3	0.68 ± 0.08^a^	3.19 ± 0.52^bc^	1.78 ± 0.15^ab^	3.86 ± 1.61^c^	4.48 ± 0.67^c^
*Total PUFAs*		*19.32 ± 2.81* ^a^	*40.30 ± 2.91* ^b^	*45.50 ± 2.95* ^b^	*46.70 ± 1.49* ^b^	*46.51 ± 1.04* ^b^

^§^Data are (expressed as) mean percentage ± SE. Values in the same row that do not share the same superscript letter are significantly different according to analysis of variance and Duncan’s post hoc procedures (*p* < .05); *nd* not detected

## Discussion

To evaluate the hypothesis that the addition of collard greens (CG), purslane (PL) or sweet potato greens (SPG) into diets with a 25:1 ω-6/ω-3 FAR will favorably modify the erythrocyte fatty acid profile, the present research was undertaken to determine the effects of the consumption of these GLVs on erythrocyte fatty acid profiles of spontaneously hypertensive rats (SHRs). Remarkably, diets supplemented with these GLVs mediated an increase in both erythrocyte mono- and polyunsaturated fatty acids, which may be beneficial in reducing the risk associated with chronic disease.

Previous research has demonstrated the ability of the ω-6/ω-3 FAR (i.e. linoleic acid:α-linolenic acid) to influence plasma docosahexaenoic acid (DHA) concentrations [27]. In a study by Ponder et al., erythrocyte DHA concentration increased by 20% when the linoleic: alpha linolenic acid (LA:ALA) ratio was decreased [28]. In addition to the ω-6/ω-3 FAR, dietary fatty acids are able to influence the erythrocyte fatty acid composition [29], which in turn is believed to be a customary indicator of long-term fatty acid intake [30]. Earlier studies found the induction of marginal changes in erythrocyte fatty acid composition by dietary fat [31]. This relationship becomes even more pronounced as the erythrocyte fatty acid composition may be an indicator of disease risk, with the PUFA content of erythrocytes being inversely associated with metabolic syndrome [32]. Reductions in erythrocyte omega-3 fatty acids have been associated with depression [33], attention deficit disorder [34] and other common mood disorders [35, 36]. Further, it has been suggested that omega-3 fatty acid deficiency may serve as a critical element in understanding the relationship between depression and cardiovascular diseases [37, 38]. Epidemiological evidence has affirmed that there exists an inverse relationship between omega-3 polyunsaturated fatty acid levels and cardiovascular disease [39–42]. However, others found omega-3 polyunsaturated fatty acid supplementation to not be associated with reductions in cardiovascular disease risk, morbidities and mortalities [43]. Further, inflammation and autoimmune diseases are believed to be exacerbated when there is insufficient omega-3 polyunsaturated fatty acids to combat the deleterious effects of pro-inflammatory cytokines and agents [44, 45].

Correcting the dietary deficiency of omega-3 fatty acids was found to favorably influence the fatty acid composition of erythrocytes in monkeys by increasing DHA content [46]. Supplementing omega-3 polyunsaturated fatty acids into the diets of pregnant women, resulted in increases in both maternal and neonatal erythrocyte concentrations of eicosapentaenoic acid (EPA) and DHA [47]. Lower levels of erythrocyte omega-3 fatty acids coupled with subsequent higher ω-6/ω-3 FARs significantly increased the risk for preeclampsia among pregnant women [48]. In addition, the source of omega-3 fatty acids was found to alter erythrocyte omega-3 fatty acid composition, with fish oil yielding a more pronounced increase in erythrocyte DHA and total omega-3 fatty acids than flaxseed oil [32].

In addition to a reduction in the ω-6/ω-3 FAR, the egg yolk omega-3 fatty acid content was increased among chickens fed diets supplemented with purslane for 84 days [49]. In another study, the inclusion of purslane and/or flaxseed oil into the diets of laying hens yielded similar results, with the purslane resulting in increased egg yolk omega-3 fatty acids [50]. Modifying the ω-6/ω-3 FAR has also been demonstrated to improve egg quality characteristics (e.g. egg weight, yolk weight, shell weight) in hens, as well as facilitating the production of eggs with higher omega-3 and other polyunsaturated fatty acid contents [51]. In this same study, greater dietary ω-6/ω-3 FARs yielded unfavorable egg characteristics that may have an adverse impact on consumer health. Increased percentages of these fatty acids may act as cellular antioxidants thwarting oxidative and inflammatory pathways implicated in disease pathogenesis [52, 53].

Lower ω-6/ω-3 FARs are desirable in reducing the risks associated with cardiovascular and other diseases [54, 55]; it has been suggested that increasing the dietary intake of omega-3 fatty acids is a viable option for optimizing tissue ω-6/ω-3 FARs [2, 56]. In the current research study a 25:1 ω-6/ω-3 FAR was examined, as this is the ratio found in the typical Western diet (i.e. American). Collard greens, purslane and sweet potato greens, incorporated into the experimental diets of the current study, have demonstrated beneficial cardioprotective, chemopreventive and anti-inflammatory effects in previous studies [57–63]. The inclusion of these GLVs resulted in increased mono- and polyunsaturated fatty acid percentages within the SHR erythrocyte, which may in turn decrease the risks associated with disease pathogenesis in an animal model predisposed to developing hypertension and other associated comorbidities.

## Conclusions

The findings of this research study provide evidence of the ability of collard greens, purslane and sweet potato greens to modify the erythrocyte fatty acid profile, even in the presence of diets with an elevated omega-6/omega-3 fatty acid ratio. The inclusion of GLVs into diets with greater than recommended omega-6/omega-3 fatty acid ratios may be useful in amending tissue and cellular fatty acid profiles in ways that may be useful in mitigating disease risk. Further, the increased PUFA and omega-3 fatty acid content of SHR erythrocytes consuming diets containing these green leafy vegetables suggest the antioxidant and erythroprotective nature of these vegetables and their potential use as a functional food with therapeutic consequences.

## Abbreviations

ω-6/ω-3 FAR: omega-6/omega-3 fatty acid ratio
ALA: Alpha linolenic acid
CG: Collard greens
CVD: Cardiovascular disease
DHA: Docosahexaenoic acid
EPA: Eicosapentaenoic acid
FAMEs: Fatty acid methyl esters
GC: Gas chromatography
GLVs: Green leafy vegetables
LA: Linoleic acid
MUFA: Monounsaturated fatty acid
PL: Purslane
PUFA: Polyunsaturated fatty acid
SFA: Saturated fatty acid
SHR: Spontaneously hypertensive rat
SPG: Sweet potato greens
